# Low-contrast detectability and potential for radiation dose reduction using deep learning image reconstruction—A 20-reader study on a semi-anthropomorphic liver phantom

**DOI:** 10.1016/j.ejro.2022.100418

**Published:** 2022-04-02

**Authors:** Tormund Njølstad, Kristin Jensen, Anniken Dybwad, Øyvind Salvesen, Hilde K. Andersen, Anselm Schulz

**Affiliations:** a Department of Radiology and Nuclear Medicine, Oslo University Hospital Ullevål, Oslo, Norway; b Department of Radiology, Haukeland University Hospital, Bergen, Norway; c Department of Diagnostic Physics, Oslo University Hospital, Oslo, Norway; d Department of Clinical and Molecular Medicine, Faculty of Medicine and Health Sciences, Norwegian University of Science and Technology (NTNU), Trondheim, Norway

**Keywords:** DLIR, deep learning image reconstruction, DLL, deep learning image reconstruction of low strength, DLM, deep learning image reconstruction of medium strength, DLH, deep learning image reconstruction of high strength, FBP, filtered back projection, IR, iterative reconstruction, Deep learning image reconstruction, CT, Low-contrast detectability

## Abstract

**Background:**

A novel deep learning image reconstruction (DLIR) algorithm for CT has recently been clinically approved.

**Purpose:**

To assess low-contrast detectability and dose reduction potential for CT images reconstructed with the DLIR algorithm and compare with filtered back projection (FBP) and hybrid iterative reconstruction (IR).

**Material and methods:**

A customized upper-abdomen phantom containing four cylindrical liver inserts with low-contrast lesions was scanned at CT dose indexes of 5, 10, 15, 20 and 25 mGy. Images were reconstructed with FBP, 50% hybrid IR (IR50), and DLIR of low strength (DLL), medium strength (DLM) and high strength (DLH). Detectability was assessed by 20 independent readers using a two-alternative forced choice approach. Dose reduction potential was estimated separately for each strength of DLIR using a fitted model, with the detectability performance of FBP and IR50 as reference.

**Results:**

For the investigated dose levels of 5 and 10 mGy, DLM improved detectability compared to FBP by 5.8 and 6.9 percentage points (p.p.), and DLH improved detectability by 9.6 and 12.3 p.p., respectively (all p < .007). With IR50 as reference, DLH improved detectability by 5.2 and 9.8 p.p. for the 5 and 10 mGy dose level, respectively (p < .03). With respect to this low-contrast detectability task, average dose reduction potential relative to FBP was estimated to 39% for DLM and 55% for DLH. Relative to IR50, average dose reduction potential was estimated to 21% for DLM and 42% for DLH.

**Conclusions**:**

Low-contrast detectability performance is improved when applying a DLIR algorithm, with potential for radiation dose reduction.

## Highlights

• Deep learning image reconstruction improves low-contrast detectability in CT.
• Performance improved with increasing strength of deep learning image reconstruction.
• Results suggest potential for CT radiation dose reduction.

## 1 Introduction

Computed Tomography (CT) has become an essential tool in modern clinical medicine [1], [2]. With widespread availability, a rapid increase in the use of CT imaging has been observed over the last decades [3]. With the associated increase in radiation exposure, the potential increased risk for radiation-induced malignancy has become a public health concern [4]. In general, the benefit of dose reduction is offset by deterioration of image quality. Thus, technological advances to reduce radiation dose without compromising image quality are aspired in clinical practice.

In CT-image reconstruction, filtered back projection (FBP) has been the dominant image reconstruction technique since the early 1970s, complemented by the first commercial iterative reconstruction (IR) algorithms in 2009 [5], [6]. Although demonstrated potential for dose reduction [7], [8], [9], recent concerns have been made that rigorous application of IR may cause a decline in low-contrast detectability due to a shift in image noise texture, particularly when radiation dose is reduced below a certain threshold [10]. Multireader phantom studies have demonstrated that IR algorithms can preserve low-contrast detectability for only modest levels of dose reduction (up to approximately 25%) [11], [12], [13], [14]. In a clinical setting, patient studies have shown that iterative reconstruction does not improve performance at moderate levels of dose reduction and that performance is deteriorated at higher levels of radiation dose reduction [15], [16].

A novel deep learning image reconstruction (DLIR) algorithm received clinical approval in 2019 (TrueFidelity, GE Healthcare, Milwaukee, WI). Other vendor-specific algorithms for deep learning image reconstruction are also developed (AiCE, Canon Medical Systems, Otawara, Japan). As explained by a technical white paper [17], having been trained with high-dose and low-dose FBP datasets across phantom and patient cases, the DLIR algorithm strives to suppress image noise without compromising image quality. The use of deep learning image reconstruction has demonstrated potential for improved image quality [18], [19], [20] and image noise reduction without shifting noise texture [21], [22], [23]. Although mathematical observer models suggest improved object detection accuracy using deep learning reconstruction [23], [24], the human observer diagnostic performance on low-contrast detection across dose levels has, to our knowledge, yet to be explored in a controlled phantom setting.

On this basis, the purpose of this study was to assess the low-contrast diagnostic performance and potential for radiation dose reduction when applying a DLIR algorithm using a customized semi-anthropomorphic upper-abdomen phantom.

## 2 Materials and methods

Institutional review board oversight was not required in this phantom-only study.

### 2.1 Phantom design

**Fig. 1:**
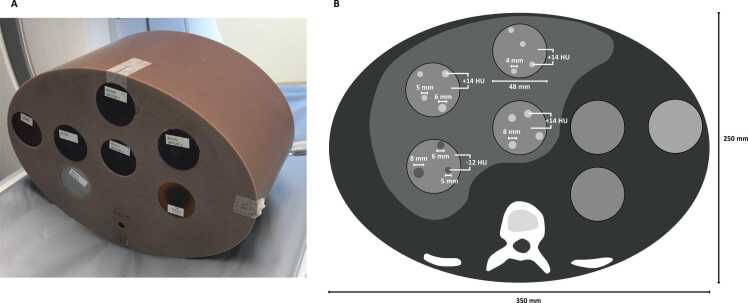
Photograph (A) and schematic (B) of semi-anthropomorphic upper-abdomen phantom applied in the study.

A customized semi-anthropomorphic upper-abdomen phantom (The Phantom Laboratory, Salem, NY) with a water equivalent diameter of 29.8 cm [25] containing four epoxy inserts with low-contrast lesions of different size and density compared to surrounding background material was applied in this study (Fig. 1).

### 2.2 Image acquisition and reconstruction

The phantom was scanned on a 16-cm multidetector CT scanner (GE Revolution; GE Healthcare; Milwaukee, WI), with scan parameters listed in Table 1, applying a range of tube currents considered clinically relevant [26]. To increase data for statistical analysis, the phantom was scanned three times for each dose level where inserts were interchanged and rotated freely about the z-axis between these scans to avoid lesion location recall bias among readers. All series were reconstructed with FBP, 50% hybrid IR (IR50; ASiR-V 50%), and DLIR of low strength (DLL; TrueFidelity Low), medium strength (DLM; TrueFidelity Medium) and high strength (DLH; TrueFidelity High) with 2.5 mm slice thickness. For reconstruction of the DLIR images, raw data were sent to the DLIR vendor (GE Healthcare) as the study was initiated prior to clinical implementation of the DLIR algorithm which is now commercially available.

**Table 1:** Scan parameters applied in study.

Scan parameter	Data
Detector collimation (mm)	80 (128 ×0.625 mm)
Tube potential (kVp)	120
Pitch	0.5
Rotation speed (seconds)	0.5
Tube current-time product (mAs)	75, 150, 225, 300 and 375
CT dose index (mGy)	5, 10, 15, 20 and 25
Matrix	512 × 512
Scan field of view	Large body
Display field of view (mm)	350
Reconstruction kernel	Standard kernel

1 Tube potential of 120 kVp chosen based on phantom size and density.

### 2.3 Reader interface and image interpretation

Twenty readers with variable level of experience (range 1–25 years) voluntarily agreed to participate in reading sessions; ten radiologists, eight medical physicists and two radiographers. Readers were unaware of study design, reconstruction algorithm applied, number of lesions and lesion configuration (i.e., size, density and location). Readings were performed independently without time constraint.

**Fig. 2:**
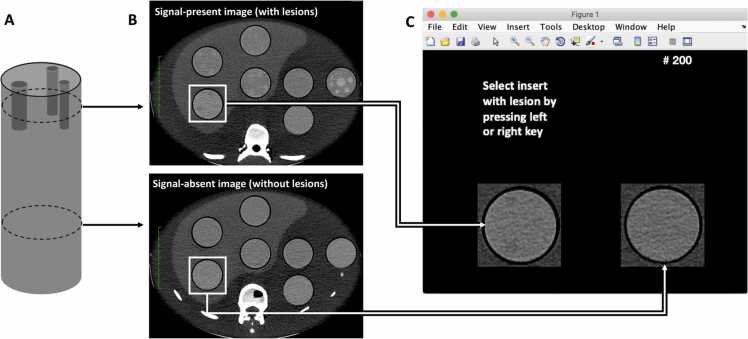
Schematic of phantom insert (A) with corresponding CT image slice (B) of insert containing lesions (signal-present image) and homogenous part of insert (signal-absent image). Screenshot of two-alternative forced choice user interface presented to readers (C).

To assess low-contrast detectability, a series of consecutive two-alternative forced choice tests were performed applying a tailored script in the MATLAB environment (version 2018a; Mathworks, Natick, MA);(Fig. 2). Readers were presented with two cropped CT-images of a specific insert, one image containing hypo- or hyperdense lesions (the signal-present image) and one image containing background noise obtained from a homogenous part of the insert (the signal-absent image). Readers were then instructed to select the image most likely to contain lesions. Images were viewed on a diagnostic display in a clinical reading room with ambient light conditions and a constant window setting with window width of 150 HU and window level of 75 HU. The signal-present image was randomly selected to be on the left or right side of the screen, and all series were presented in a random order. Two different signal-present images were extracted from each scan. Thus, each reader was presented with a total of 600 pairwise image comparisons; 2 signal-present images x 3 scans x 4 inserts x 5 dose levels x 5 reconstruction algorithms. Finally, to reduce the potential effect of inhomogeneity in background noise mimicking a lesion, the signal-absent image was obtained from two separate z-locations of the homogenous part of the insert.

### 2.4 Detectability and dose reduction potential

Detectability was defined as the fraction of correctly selected images containing lesions, and was assessed separately for each dose level and reconstruction algorithm across readers. The dose reduction potential of the DLIR algorithm was defined as the reduction in dose level in which detectability was equal to that of FBP or IR50. To estimate detectability at dose levels not explicitly evaluated in the study setup, observer data was fitted to a mathematical model using the least squares approach in the Matlab software. With the assumption of detectability of 0.5 with dose level approaching zero (i.e., randomly guessing) and converging towards 1 with increasing dose level, the observer scores were modeled according to the equation:

(1) $$\begin{array}{c} D=\frac{1}{2}\left[1+\mathrm{erf}\left(\frac{\alpha ∙d^{\beta }}{2}\right)\right]\; \end{array}$$

where *D* is detectability, *erf* is the error function, *d* is the dose level, and α and β are constant fitting parameters [9]. In this mathematical model, detectability is modelled to 0.5 when dose level, d, approaches zero, as erf(0) = 0. With increasing dose level, the modelled detectability converges towards 1, as erf(∞) = 1. Dose reduction potential was estimated separately for each strength of DLIR, and estimated separately for each investigated dose level. Subgroup analyses were performed comparing radiologist to non-radiologist reader scores.

### 2.5 Statistical analysis

Comparisons of reader detectability scores across reconstruction algorithms were performed applying a pairwise student’s t-test at the 95% significance level under the null hypothesis that there was no difference in detectability between reconstruction algorithms. Adjusted odds ratios (OR) for correctly selecting the signal-present image (i.e., with low-contrast lesions) were estimated using mixed logistic regression. For this, reconstruction algorithm, dose level and lesion type were included as fixed effects whereas reader and the combination of scan number and CT image slice were random effects. Model estimates were computed using R statistical software (version 3.0.4, R Foundation for Statistical Computing, Vienna, Austria, (https://www.r-project.org/) applying the lme4 package [27].

## 3 Results

**Fig. 3:**
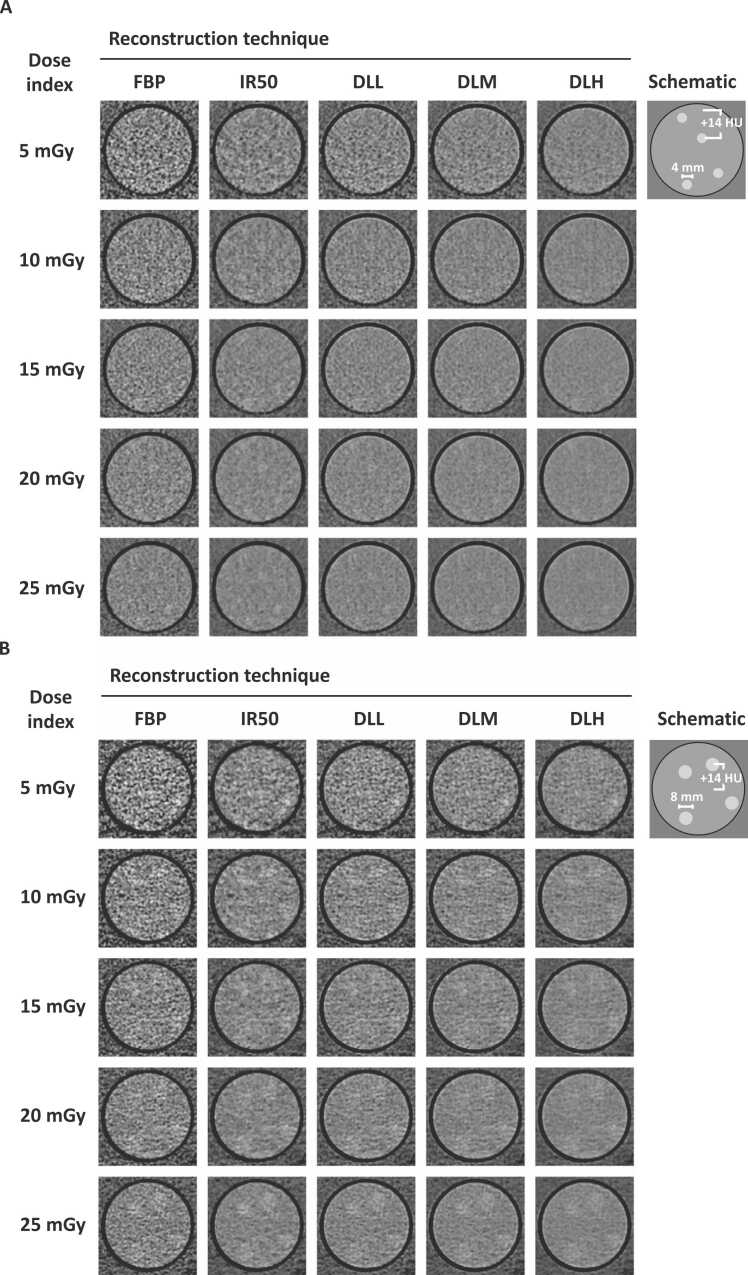
Example CT-images of phantom insert with small ~4 mm lesions (A) and large ~8 mm lesions (B) for each reconstruction algorithm and dose level with schematic of ground truth.

Example images of inserts across dose levels and reconstruction algorithms containing small and large lesions are presented in Fig. 3A and Fig. 3B, respectively. From the ensemble of 20 readers, a total of 12000 data points were obtained. One response was discarded due to reader misclassification, and thus 11,999 data points were included for the final analyses.

**Fig. 4:**
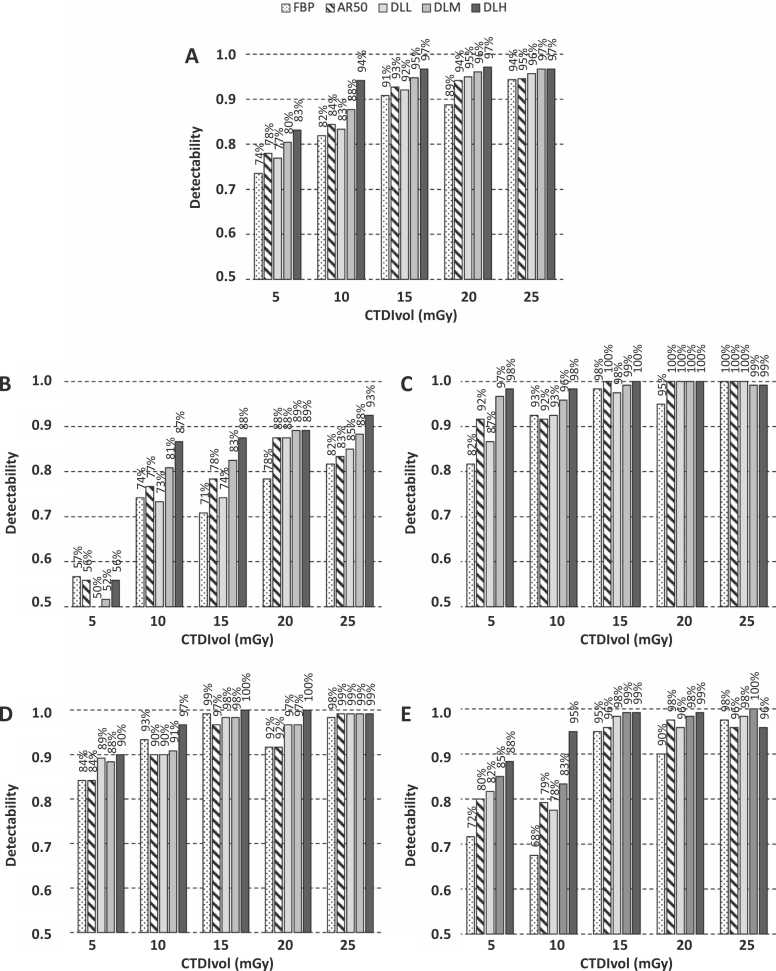
Results from reader sessions. Bar charts show average reader detectability scores at an aggregate level (A) and separately for each lesion type with 4 mm hyperdense lesions (B), 6 mm hyperdense lesions (C), 8 mm hyperdense lesions (D) and 8 mm hypodense lesions (E). Images were reconstructed with filtered back projection (FBP), 50% hybrid iterative reconstruction (IR50), and deep learning image reconstruction (DLIR) of low strength (DLL), medium strength (DLM) and high strength (DLH).

Detectability at an aggregate level for each reconstruction algorithm and subgroup analysis per insert type is presented in Fig. 4. Performance ranged from approximately 50% (i.e., random guess) for the insert with small lesions imaged at 5 mGy dose level to 100% for the insert with large lesions imaged at 25 mGy dose level. When comparing the DLIR algorithms to FBP, detectability was significantly improved for images reconstructed with DLM and DLH for all dose levels (all p < .003). The largest improvement in detectability was observed for the lower dose levels, where DLM improved detectability by 6.9 p.p. at the 5 mGy dose level (95% CI 2.1–11.7 p.p., p = .007) and 5.8 p.p. at the 10 mGy dose level (95% CI 2.3–9.4 p.p., p = .003). Correspondingly, DLH improved detectability by 9.6 p.p. at the 5 mGy dose level (95% CI 3.5–15.6 p.p., p = .004) and 12.3 p.p. at the 10 mGy dose level (95% CI 8.1–16.5 p.p., p < .001). Compared to IR50, detectability was significantly improved for images reconstructed with DLH for the 5, 10 and 15 mGy dose level (p < .03), with the highest observed difference at the 10 mGy dose level (+9.8 p.p. improvement in detectability, 95% CI 4.8–14.8 p.p., p = .001). An overview of differences in detectability per dose level and reconstruction algorithm is presented in Table 2.

**Table 2:** Differences in detectability scores in percentage points (p.p.) for images reconstructed with DLIR of various strengths relative to hybrid IR and FBP.

		DLL		DLM		DLH	
Reference	CT dose index, mGy	Difference in detectability, p.p. (95% CI)	p-value	Difference in detectability, p.p. (95% CI)	p-value	Difference in detectability, p.p. (95% CI)	p-value
FBP	5	+ 3.3 (−2.9, +9.6)	*.28*	+ 6.9 (+2.1, +11.7)	.007	+ 9.6 (+3.5, +15.6)	.004
	10	+ 1.5 (−2.8, +5.7)	*.48*	+ 5.8 (+2.3, +9.4)	*.003*	+ 12.3 (+8.1, +16.5)	< 0.001
	15	+ 1.3 (−2.0, +4.5)	*.43*	+ 4.0 (+1.5, +6.5)	*.003*	+ 5.8 (+3.2, +8.5)	< 0.001
	20	+ 6.3 (+2.7, +9.8)	*.002*	+ 7.3 (+4.5, +10.1)	*< 0.001*	+ 8.3 (+4.8, +11.9)	< 0.001
	25	+ 1.3 (−0.3, +2.8)	*.11*	+ 2.3 (+0.2, +4.3)	*.03*	+ 2.3 (+0.6, +4.0)	.01
IR50	5	-1.0 (−5.7, +3.6)	.64	+ 2.5 (−0.9, +5.9)	.14	+ 5.2 (+0.7, +9.7)	.03
	10	-1.0 (−5.3, +3.2)	.61	+ 3.3 (−0.6, +7.3)	.09	+ 9.8 (+4.8, +14.8)	.001
	15	-0.6 (−3.3, +2.0)	.62	+ 2.1 (−0.9, +5.1)	.16	+ 4.0 (+1.7, +6.2)	.001
	20	+ 0.8 (−2.0, +3.7)	.55	+ 1.9 (−0.8, +4.6)	.17	+ 2.9 (−0.8, +6.6)	.11
	25	+ 1.0 (−0.8, +2.9)	.26	+ 2.1 (−0.3, +4.5)	.09	+ 2.1 (−0.3, +4.5)	.09

P-values for difference by pairwise student’s t-test.

DLH = deep learning image reconstruction of high strength, DLL = deep learning image reconstruction of low strength, DLM = deep learning image reconstruction of medium strength, FBP = filtered back projection, IR50 = 50% hybrid iterative reconstruction.

Results from mixed logistic regression are presented in Table 3. Adjusted for lesion type and dose level, odds ratio (OR) for correctly selecting the signal-present CT image was significantly improved for all strengths of DLIR compared to FBP, with OR estimated to 1.41 for DLL (95% CI 1.18–1.75, p < .001), 2.05 for DLM (95% CI 1.66–2.52, p < .001) and 3.20 for DLH (95% CI 2.55–4.02, p < .001). Compared to IR50, OR for correctly selecting the signal-present CT image was significantly improved for the higher strengths of DLIR, with OR estimated to 1.42 for DLM (95% CI 1.15–1.77, p = .001) and 2.23 for DLH (95% CI 1.76–2.81, p < .001).

**Table 3:** Results from mixed logistic regression with estimated adjusted odds ratio (OR) for correctly selecting signal-present CT image.

Fixed effects variable	Adjusted OR with FBP as reference (95% CI)	P-value	Adjusted OR with IR 50 as reference (95% CI)	P-value
Intercept	0.44 (0.22–0.86)	.02	0.63 (0.32–1.24)	.18
Dose level	1.14 (1.10–1.18)	< 0.001	1.14 (1.10–1.18)	< 0.001
Lesion type				
4 mm hyperdense lesions	Reference		Reference	
6 mm hyperdense lesions	15.6 (8.06–30.23)	< 0.001	15.6 (8.06–30.23)	< 0.001
8 mm hyperdense lesions	8.81 (4.67–16.62)	< 0.001	8.81 (4.67–16.62)	< 0.001
8 mm hypodense lesions	4.07 (2.21–7.49)	< 0.001	4.07 (2.21–7.49)	< 0.001
Reconstruction algorithm				
FBP	Reference	–	0.70 (0.57–0.85)	< 0.001
IR50	1.44 (1.18–1.75)	< 0.001	Reference	–
DLL	1.41 (1.15–1.71)	< 0.001	0.98 (0.80–1.20)	.83
DLM	2.05 (1.66–2.52)	< 0.001	1.42 (1.15–1.77)	.001
DLH	3.20 (2.55–4.02)	< 0.001	2.23 (1.76–2.81)	< 0.001

DLH = deep learning image reconstruction of high strength, DLL = deep learning image reconstruction of low strength, DLM = deep learning image reconstruction of medium strength, FBP = filtered back projection, IR50 = 50% hybrid iterative reconstruction.

**Fig. 5:**
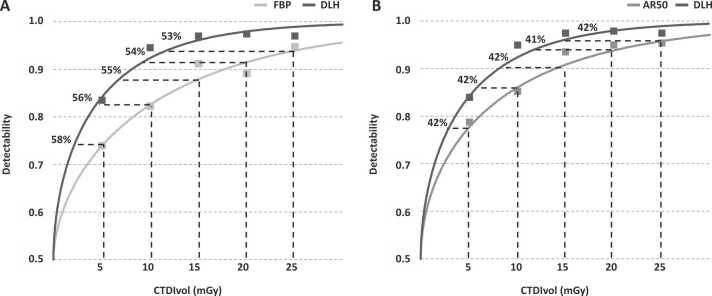
Plot of average low-contrast detectability scores for images reconstructed with deep learning image reconstruction of high strength (DLH) and filtered back projection (FBP);(A) and 50% hybrid iterative reconstruction (IR50);(B) for each investigated CT dose index (CTDIvol). Curves fitted to observer data allow for estimation of the potential reduction in dose level for DLH while maintaining comparable level of detectability to FBP and IR50 for each investigated dose level (dashed lines).

Dose reduction potential with FBP as reference was estimated to an average of 20% for DLL (range 17–22%), 39% for DLM (range 36–42%) and 55% for DLH (range 53–58%);(Table 4). With IR50 as reference, dose reduction potential was estimated to 21% for DLM (range 19–22%) and 42% for DLH (range 41–43%). Fig. 5 illustrates the dose reduction potential of DLH based on the detectability scores for DLH and IR50 across the investigated dose levels.

**Table 4:** Estimated dose reduction potential for hybrid IR and DLIR of various strengths relative to FBP.

	Dose level (mGy)				Dose reduction potential			
Reference FBP dose (mGy)	IR50	DLL	DLM	DLH	IR50	DLL	DLM	DLH
5	3.6	4.2	2.9	2.1	29%	17%	42%	58%
10	7.5	8.1	6.0	4.4	25%	19%	40%	56%
15	11.7	12.0	9.3	6.8	22%	20%	38%	55%
20	15.9	15.7	12.5	9.2	20%	21%	37%	54%
25	20.3	19.5	15.9	11.7	19%	22%	36%	53%

Estimated dose level with comparable detectability performance and implied dose reduction potential for hybrid IR and DLIR of various strengths relative to FBP based on a mathematical model fitted to low-contrast detectability observer data.

DLH = deep learning image reconstruction of high strength, DLL = deep learning image reconstruction of low strength, DLM = deep learning image reconstruction of medium strength, FBP = filtered back projection, IR50 = 50% hybrid iterative reconstruction.

When comparing radiologists (n = 10) to non-radiologists (n = 10), detectability scores were at an aggregate level slightly higher for radiologists (+2.9 p.p.). However, there was no trend for radiologists performing better for a specific dose level or reconstruction technique (Supplementary Table 1). Dose reduction potential using radiologist scores only and with FBP as reference was estimated to an average of 18%, 34% and 53% for DLL, DLM and DLH, respectively. With IR50 as reference, dose reduction potential was estimated to an average of 21% and 38% for DLM and DLH, respectively.

## 4 Discussion

This tailored phantom study demonstrates that CT images reconstructed with a DLIR algorithm can improve low-contrast detectability relative to conventional FBP and hybrid IR, and that this improvement in detectability is dependent on algorithm strength. Based on reader detectability scores, average dose reduction potential for DLIR of high strength was estimated to 55% compared to FBP and 42% compared to 50% hybrid IR.

Fueled by increased availability, a rapid increase in the use of CT imaging has been observed over the last decades and the associated increase in radiation exposure has become a public health concern [3], [4]. Although CT provides tremendous benefit to patient care when used for appropriate indications, efforts to keep radiation dose delivered to the patient as low as reasonably achievable have been widely endorsed (i.e., the ALARA principle) [28]. However, decreasing radiation dose is traditionally offset by a deterioration in image quality, driven by an increase in image noise [29]. Detection and characterization of focal liver lesions can be a challenging diagnostic task in abdominal imaging, with decisive implication for patient care in an oncological setting [30]. This low-contrast task is highly sensitive to image noise – especially if suboptimal dose levels have been applied during image acquisition [30], [31]. Recent reports have shed light on potential disadvantages of IR algorithms where dose reduction potential may be limited by deterioration in image quality primarily affecting low-contrast tasks, such as obscuring small liver lesions [10]. Thus, novel methods to reduce CT image noise are aspired in clinical practice in pursuit of dose reduction.

Several studies have investigated the qualitative image quality of DLIR algorithms. Phantom studies have demonstrated that a DLIR algorithm can achieve robust noise reduction without deteriorating changes in image noise texture, although demonstrating somewhat degraded low-contrast spatial resolution [21], [22]. A recent phantom study by Racine al. investigating low-contrast detectability using channelized Hotelling observer as a surrogate marker for human observers estimated the dose reduction potential to 25% for DLM to and 33% for DLH compared to 60% hybrid IR [24]. The current study is thus an important supplement, demonstrating improved low-contrast detectability performance by human observers. In a clinical setting, the DLIR algorithm has demonstrated improved perceived overall image quality when compared to standard IR at standard dose abdominal CT [19], [20]. A study on images of pediatric patients reconstructed with a DLIR algorithm from a different vendor (AiCE, Canon Medical Systems) found that significant dose reduction was possible without sacrificing image quality [23]. A study on low-dose abdominal CT applying the same vendor-specific algorithm found that deep learning reconstruction improves overall image quality and lesion detection compared to FBP and IR images [32]. It should be noted that the AiCE algorithm is trained with model-based IR images, and results are not necessarily transferrable across vendors. Thus, it is important to take into account how the different algorithms are trained when evaluating and comparing vendor-specific DLIR algorithms.

Interestingly, radiologist detectability scores were significantly, albeit only slightly, higher than non-radiologists. Subgroup analysis of results based in radiologist scores were important as radiologists assess the images in clinical practice. However, selecting a signal-present image in a two-alternative forced choice approach does not take into account the diagnostic considerations a radiologist is expected to take when assessing focal liver lesions. Thus, studies incorporating detectability in combination with assessment of diagnostic confidence in a clinical setting with clinicopathological correlation are important supplements to this study in further exploring the dose reduction potential of deep learning based CT image reconstruction algorithms.

This study is not without limitations. First, only one specific scanner and one vendor-specific DLIR algorithm was applied, and results are not necessarily transferrable across vendors. Second, this study only compared one level of IR where several blends of hybrid-IR could have been explored. However, there is a practical balance between the number of metrics explored in such a human observer setting. This study was designed to explore different strengths of DLIR, and compare with standardly applied hybrid IR at our institution in addition to FBP as this vendor-specific DLIR algorithm is trained on FBP datasets. Third, this study lacks validation in a clinical setting, although the use of a semi-anthropomorphic upper-abdomen phantom containing lesions with a variation in size arguably mitigates this limitation.

In conclusion, this study demonstrates that low-contrast detectability is improved when applying a novel DLIR algorithm, and that this improvement is dependent on algorithm strength. The dose reduction potential based on detectability performance for DLIR of high strength was estimated to 55% relative to FBP and 42% relative to hybrid IR. The results in this study can serve as a basis for clinical studies to further investigate the diagnostic performance and dose reduction potential of DLIR.

## Funding sources

This research did not receive any specific grant from funding agencies in the public, commercial, or not-for-profit sectors.

## CRediT authorship contribution statement

**Tormund Njølstad**: Conceptualization, Methodology, Investigation, Writing – original draft. **Kristin Jensen**: Data Curation, Investigation, Writing – review & editing. **Anniken Dybwad**; Data Curation, Investigation, Writing – review & editing. **Øyvind Salvesen**; Investigation, Writing – review & editing. **Hilde K. Andersen**: Investigation, Supervision, Writing – review & editing. **Anselm Schulz**: Conceptualization, Writing – review & editing, Supervision, Project administration.

## Declaration of Competing Interest

The authors declare the following financial interests/personal relationships which may be considered as potential competing interests:This study is part of ongoing research at the Oslo University Hospital CT Research and Technology Group. Oslo University Hospital has institutional research agreements with GE Healthcare and The Phantom Laboratory, among others. The authors of this article had complete control of data that might have presented a conflict of interest throughout the study period, and the decision to publish has been at the sole discretion of the authors. The authors state no individual conflicts of interests.
