# Supplementary material for: Low-contrast detectability and potential for radiation dose reduction using deep learning image reconstruction—A 20-reader study on a semi-anthropomorphic liver phantom
Source: Eur J Radiol Open. 2022 Apr 2;9:100418. doi: 10.1016/j.ejro.2022.100418 (PMC8980706; doi:10.1016/j.ejro.2022.100418)
Supplement: Supplementary file 1 — Supplementary material [file mmc1.docx]

**Supplementary Table 1**: Differences in detectability scores (average detectability) in percentage points (p.p.) between radiologists (n=10) and non-radiologists (n=10).

| Dose index | Reconstruction algorithm | Average detectability score radiologists | Average detectability score non-radiologists | Mean difference (95% CI) | P-value ^1^ |
| --- | --- | --- | --- | --- | --- |
| 5 mGy | FBP | 0.77 | 0.70 | 0.07 (-0.02, 0.17) | .13 |
|  | IR50 | 0.79 | 0.77 | 0.02 (-0.06, 0.10) | .67 |
|  | DLL | 0.79 | 0.75 | 0.04 (-0.05, 0.12) | .36 |
|  | DLM | 0.80 | 0.81 | -0.02 (-0.08, 0.05) | .59 |
|  | DLH | 0.85 | 0.81 | 0.05 (-0.02, 0.11) | .17 |
| 10 mGy | FBP | 0.85 | 0.79 | 0.05 (-0.02, 0.13) | .14 |
|  | IR50 | 0.88 | 0.81 | 0.06 (-0.04, 0.16) | .20 |
|  | DLL | 0.87 | 0.80 | 0.07 (-0.03, 0.16) | .15 |
|  | DLM | 0.92 | 0.84 | 0.08 (0.01 0.15) | .03 |
|  | DLH | 0.94 | 0.94 | 0.00 (-0.05,0.05) | .99 |
| 15 mGy | FBP | 0.94 | 0.88 | 0.07 (0.02 0.11) | .004 |
|  | IR50 | 0.93 | 0.92 | 0.01 (-0.03,0.06) | .55 |
|  | DLL | 0.92 | 0.93 | -0.01 (-0.05,0.03) | .67 |
|  | DLM | 0.96 | 0.93 | 0.03 (-0.02,0.07) | .19 |
|  | DLH | 0.98 | 0.96 | 0.02 (-0.01,0.05) | .26 |
| 20 mGy | FBP | 0.89 | 0.88 | 0.01 (-0.06,0.08) | .80 |
|  | IR50 | 0.98 | 0.90 | 0.07 (0.00 0.15) | .049 |
|  | DLL | 0.98 | 0.92 | 0.06 (0.02 0.10) | .01 |
|  | DLM | 0.98 | 0.94 | 0.05 (0.00 0.09) | .03 |
|  | DLH | 0.98 | 0.96 | 0.02 (0.00 0.04) | .12 |
| 25 mGy | FBP | 0.95 | 0.94 | 0.01 (-0.02,0.04) | .38 |
|  | IR50 | 0.94 | 0.95 | -0.01 (-0.05,0.04) | .69 |
|  | DLL | 0.96 | 0.95 | 0.00 (-0.02,0.03) | .75 |
|  | DLM | 0.96 | 0.97 | -0.01 (-0.04,0.02) | .54 |
|  | DLH | 0.96 | 0.97 | -0.01 (-0.04,0.02) | .54 |

1 P-value by student’s t-test

DLL = deep learning image reconstruction of low strength, DLM = deep learning image reconstruction of medium strength, DLH = deep learning image reconstruction of high strength, FBP = filtered back projection, IR50 = 50% hybrid iterative reconstruction.
